# Colour Change of Sustainable Concrete Containing Waste Ceramic and Hybrid Fibre: Effect of Temperature

**DOI:** 10.3390/ma15062174

**Published:** 2022-03-15

**Authors:** Hadee Mohammed Najm, Ominda Nanayakkara, Mahmood Ahmad, Mohanad Muayad Sabri Sabri

**Affiliations:** 1 Department of Civil Engineering, Zakir Husain Engineering College, Aligarh Muslim University, Aligarh 202002, India; 2 Department of Civil Engineering, Xi’an Jiaotong-Liverpool University, Suzhou 215000, China; ominda.nanayakkara@xjtlu.edu.cn; 3 Department of Civil Engineering, Faculty of Engineering, International Islamic University Malaysia, Jalan Gombak 50728, Selangor, Malaysia; ahmadm@iium.edu.my; 4 Department of Civil Engineering, University of Engineering and Technology Peshawar (Bannu Campus), Bannu 28100, Pakistan; 5 Peter the Great St. Petersburg Polytechnic University, 195251 St. Petersburg, Russia; mohanad.m.sabri@gmail.com

**Keywords:** waste ceramic optimal concrete, elevated temperature, hybrid concrete, polyvinyl alcohol fibre, crimped steel fibre

## Abstract

Construction and demolition (C&D) waste was considered as zero value materials and, as a result, most C&D waste materials ended up in landfills, eventually constituting environmental issues. Therefore, it is important to explore the potential of such C&D waste materials for concrete production. Thus, this research effort aims to find a new method to improve sustainable concrete properties exposed to elevated temperatures at the lowest costs and identify the relationship between temperature change and a change in colour in a heat-exposed concrete structure. Therefore, this study explored the behaviour of three types of concrete: plain concrete (PC), waste ceramic optimal concrete (WOC), and waste ceramic optimal concrete reinforced by hybrid fibre (WOC-Hybrid) in ambient and elevated temperature. The study shows that colour change in a concrete structure exposed to high temperature has a consistent relationship, where it has been found that the colour alteration is of great interest because its appearance usually coincides with the onset of a significant loss of concrete strength as a result of elected temperatures. Overall, it can be considered that waste ceramic materials reinforced by hybrid fibres can be used for concrete production, and by doing so, the negative impact of these wastes on the environment can be controlled as well as fortify the mechanical properties.

## 1. Introduction

Resistance of concrete structures to rising temperatures is a primary global concern today. Therefore, it requires a solid and unified effort to arrive at a heat-resistant economic concrete using readily available resources. A promising source of low-cost materials is the growing waste generated due to excessive urbanisation, global economic development, and rapid industrialisation. Casares, M. L. et al. [1] noted that “waste materials contain harmful to the environment and humans’ toxic chemicals and traditional waste management practices commonly employed generally increase environmental contamination”. Therefore, the aim is to find solutions to the increasing quantity of waste dumped into the natural environment by finding the optimum use of waste materials. One such process uses concrete materials with particular characteristics, reducing prices and improving their characteristics [2].

When conventional concrete is exposed to heat, exceptionally elevated temperatures lose significant strength or even wholly degrade. The presence of limestone rocks and ordinary cement in concrete is the main reason for the cracks and the release of certain gases when exposed to elevated temperatures. However, limestone is the primary reason that contributes to this process. Ordinary rocks are primarily composed of lime. Regular lime (CaCO_3_) degrades to form quicklime (CaO), which is extremely hydrophilic. When lime comes into contact with water or humidity in the air, it will absorb a large amount of water. Subsequently, its volume will increase. This volume increase results in cracks in the rocks, thus causing the concrete to collapse [3]. As an alternative to such heavy reliance on limestone concrete mixes, previous research has used various waste materials, such as ceramic wastes, to increase the material’s heat resistance. Ceramic is a material that resists pressure, humidity, and high temperatures; thus, it is used in wall and floor façades; as a result, it is also effective at improving the properties of concrete [4,5,6].

Najm and Ahmad [7] proved that the concrete mixes containing ceramic waste materials such as cement and aggregate replacement have been shown to exhibit enhanced mechanical properties of concrete. Moreover, Kulovaná, T. et al. [8] proved that “ceramics ameliorate concrete resistance to chemical and heat attacks while decreasing concrete water absorption”. Thus, in general, studies indicate that using ceramic waste in concrete is feasible, though there may be some differences in the concrete’s properties. Furthermore, ceramics increase the resistance of concrete to chemical and thermal attacks while decreasing its water absorption [9].

Recent research has focused on using ceramic wastes of various sizes as a whole or partial replacement of concrete aggregates to reduce the adverse environmental effects of waste ceramics [10]. In addition, other studies have shown that powdered ceramic waste can be used in place of Portland cement as a pozzolanic material [11]. In general, studies indicate that using ceramic waste in concrete is feasible, though there may be some differences in the concrete’s properties. In the same regard of the effect of elevated temperatures on concrete materials, ceramic waste had a better thermal resistance than plain concrete when exposed to higher temperatures [12]. Moreover, using ceramic waste materials in both forms of binder and aggregate significantly improved the mechanical properties of concrete and gave it better resistance to elevated temperatures. In addition, the microstructure of the concrete was improved if ceramic materials were used [13]. One reason for concrete damage at elevated temperatures is thermal stress concentration due to heterogeneous heat distribution in concrete. Researchers used metallic and non-metallic fibre in concrete exposed to high temperatures to counteract this effect; for example, Gao, D. et al. [14] studied the effect of PP fibres in dosage 0.5, 1, 1.5, and 2% (by volume) on tensile strength behaviour under elevated temperatures 100 °C, 450 °C, and 650 °C. Additionally, the utilisation of ceramic waste materials with metallic or non-metallic fibre along with other aggregates may affect the performance of the concrete. Hosseini et al. [13] concluded that “the used non-metallic fbres in the production of green concrete composite exposed at elevated temperatures (200, 400, 600 and 800 °C) for 1 h, where the fire resistance and residual strength of the concrete mixed with non-metallic fibres increased at elevated temperatures. Furthermore, the role of non-metalic fibres is discussed through the microstructural analysis and fibre–matrix interactions as a function of heat treatment [15]”. Han, C. G. et al. [16] noted that “the non-metallic fibers reinforced concrete has much better resistance to thermal spalling than the concrete without fibers because of the melting and ignition of non-metallic fibers. Consequently, the pores formed to expand to form micro-cracks, connecting the existing capillary pores to provide channels for water vapor to escape”.

Many concrete structures are prone to crack and collapse after exposure to high temperatures, especially over time because of limestone in the mix. Therefore, such damage to concrete may be avoided if ceramic waste materials replace natural materials. In addition, hybrid fibres can significantly reduce the risk of concrete cracking, increase thermal conductivity, and reduce the concrete’s internal stresses. Najm and Ahmad [17] used ceramic waste materials with hybrid fibres in the production of sustainable concrete exposed to elevated temperatures (100, 200, and 300 °C) for 1 h. It was concluded that hybrid fibre concrete (WOC-Hybrid) and waste ceramic concrete (WOC) performs better than plain concrete because of an increase in the transport properties of heated material (concrete with ceramic tiles has lower thermal conductivity than concrete with natural aggregates). In addition, heat transmits more uniformly in the concrete reinforced with hybrid fibres.

Thus, among the previous studies, aside from the combination of hybrid fibre and ceramic wastes for making WOC and WOC-Hybrid concrete, there are no available data on the effect of thermal conditions on the physical and mechanical properties of these novel mixtures which can be considered an indication of the novelty of the present study. Therefore, the current work presents a preliminary study on the effect of ceramic material (ceramic powder, fine and coarse ceramic aggregate) and hybrid fibre (PVA and CR) on the colour change of WOC and Hybrid-WOC subjected to elevated temperatures. Several laboratory experiments have been conducted to investigate WOC and Hybrid-WOC’s behaviour. In turn, it provides a basis for further research on WOC and Hybrid-WOC concrete and its potential applications.

In the present study, the authors propose a new concrete mix named WOC and Hybrid-WOC. Many considerations have been coined based on the new generation of concrete: (1) PVA + crimped steel fibre is used to improve the performances of concrete both before and after exposure to elevated temperatures. (2) The inclusion of ceramic waste material is mainly for environmental protection and ceramic tiles have lower thermal conductivity and better fire resistance after exposure to high temperatures. (3) There is beneficial interaction between hybrid fibre (PVA+CR) and ceramic, so resistance to elevated temperatures makes Hybrid-WOC concrete useful in fire-resistant structures.

Finally, the study’s main contribution is to evaluate the potential use of colour change analysis in fire-damaged concrete investigations. Concrete samples were subjected to steady-state heating to be analysed for colour changes. This colour alteration is of great interest because its appearance usually coincides with the onset of a significant loss of concrete strength as a result of heating. Furthermore, we investigate the fundamental properties of a new generation of concrete (WOC and WOC-Hybrid) as sustainable concrete in ambient and under elevated temperatures.

This article is divided into the following sections: application of a colour change in fire sciences is briefly introduced in Section 2. In Section 3, experimental work is described in detail, including a description of materials used, preparation of specimens, concrete mix design, test procedures and colour measurement method. In Section 4, results and discussion of the experimental work are analysed as the effect of elevated temperatures on concrete colour changes of new generation concrete (WOC and WOC-Hybrid Fibres) under elevated temperatures. Finally, conclusions are given in Section 5

## 2. Application of Colour Change in Fire Sciences

Colourimetry and the physical phenomenon of solid colour change due to heating are used in many fields of fire science research. It has been documented in the wildfire literature that physical and chemical changes at the soil surface during a fire can result in colour shifts [18]. Colour analysis was used to estimate maximum temperature and exposure time. The CR-300 colourimeter and the Munsell colour system were used in research by Ketterings, Q. M. and Bigham, J. M. [18] to measure changes in the chromas and hues of heated soils. It is well known that the colour of building materials can change when they are exposed to high temperatures, as demonstrated in the research carried out by the Building Research Establishment, summarised in Chakrabarti, B., Yates, T., and Lewry, A. [19], and significant colour changes in stones containing minerals with iron compounds were reported. Changes in the colour of historical monuments built of sandstone when exposed to fire were also confirmed recently by [20].

Temperature and the type of aggregate used in the mix can influence how much colour changes in heated concrete. Concrete containing silicate aggregates (quartz, flint) will turn red when heated to a temperature between 300 °C and 600 °C, according to the research of Short, N. R. et al. [21]. The cement matrix turns whitish-grey at temperatures between 600 °C and 900 °C while heating it to between 900 °C and 1000 °C results in a buff colour. Comparisons with concrete that has not been exposed to high temperatures can help identify temperature-induced colour changes in concrete.

An inherently subjective visual colour analysis is currently used to make a rough estimate of the temperature exposure of the concrete. Felicetti R. [22] has used various colour description techniques to describe concrete colour changes precisely. Researchers Colombo, M. and Felicetti, R. [23] used an infrared spectrophotometer to measure changes in colour on concrete samples. Another research study [24] used spectrophotometers to examine the colour of heated concrete ground into powder. Colour analysis software and a polarising microscope were used by Short, N. R. et al. [21] to observe samples. In Felicetti R. [22], a general-purpose digital camera was used to photograph and analyse concrete colour changes as a function of temperature. On the other hand, this method necessitates consistent lighting, which can be challenging, and the camera’s white balance must be adjusted in a dark room.

To be sure, colour shifts are unrelated to concrete’s mechanical and physical properties, but they do serve as a gauge for the fire’s temperature rise. Several techniques should be used together in practice for a complete and accurate picture of the damage done to the concrete member [25]. A thorough assessment must be carried out to make the right decision on whether or not to strengthen or repair fire-damaged construction elements.

For assessing concrete damage caused by fire, the above-mentioned all agree that the material colour change is a valuable indicator of maximum temperature exposure.

## 3. Methods

### 3.1. Description of Materials Used

**Figure 1:**
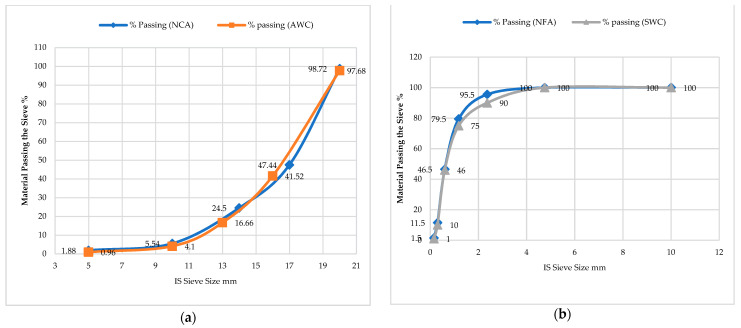
Particle size distribution of (**a**) natural coarse aggregate and ceramic coarse aggregate; (**b**) natural fine aggregate and ceramic fine aggregate.

**Figure 2:**
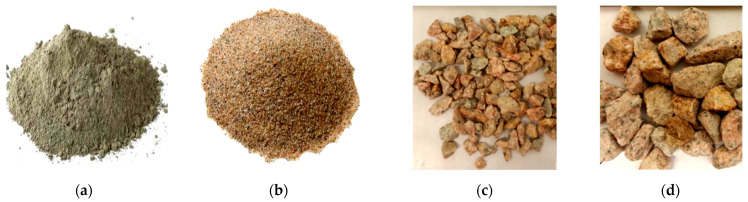
(**a**) Commercial cement; (**b**) river sand; (**c**) natural coarse aggregates (NCA)—10 mm; (**d**) natural coarse aggregates (NCA)—20 mm.

**Figure 3:**
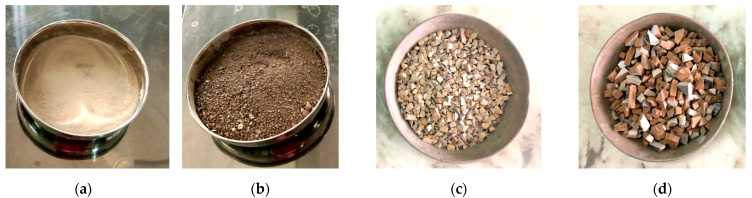
(**a**) Waste ceramic cement (C_WC_)—75 µm; (**b**) waste ceramic sand (S_WC_)—4.75 mm (**c**) waste ceramic aggregate (A_WC_)—10 mm; (**d**) waste ceramic aggregate (A_WC_)—20 mm.

**Figure 4:**
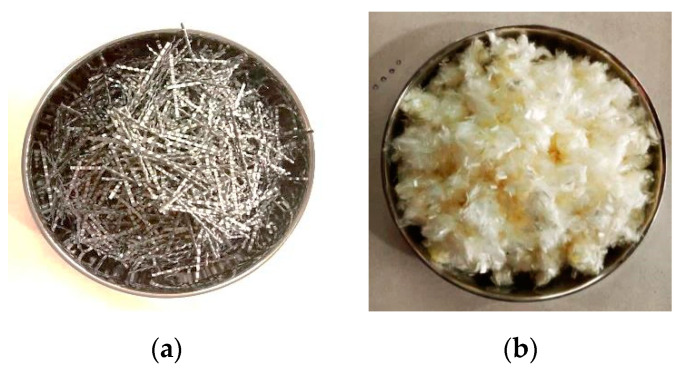
Metallic and non-metallic fibre: (**a**) crimped steel fibre (CR) (60 mm); (**b**) polyvinyl alcohol fibre (PVA) (12 mm).

Locally available materials such as ordinary Portland cement (OPC) 43-grade, natural coarse aggregate (NCA)/stones, coarse river sand, and ceramic floor tiles were used in the experimental work (in place of cement, sand, and coarse aggregate), as shown in Figure 1. Aligarh’s Ceramic Stores provided the waste ceramic floor tiles used in this project. Ceramic tiles were cleaned and dusted off before being hammered into various sizes: 20 mm and 10 mm (waste ceramic aggregate-A_WC_), 4.75 mm (waste ceramic sand-S_WC_), and 75 μm (waste ceramic cement-C_WC_) as shown in Figure 2. The physical properties of ceramic waste floor tiles (A_WC_, S_WC,_ and C_WC_), OPC, river sand, and natural aggregate are shown in Table 1 and Figure 3 and Figure 4. Types of fibre-reinforcements have been used, namely crimped steel fibre (CR) and polyvinyl alcohol fibre (PVA), shown in Figure 3, where its properties are shown in Table 2.

### 3.2. Preparation of Specimens

**Figure 5:**
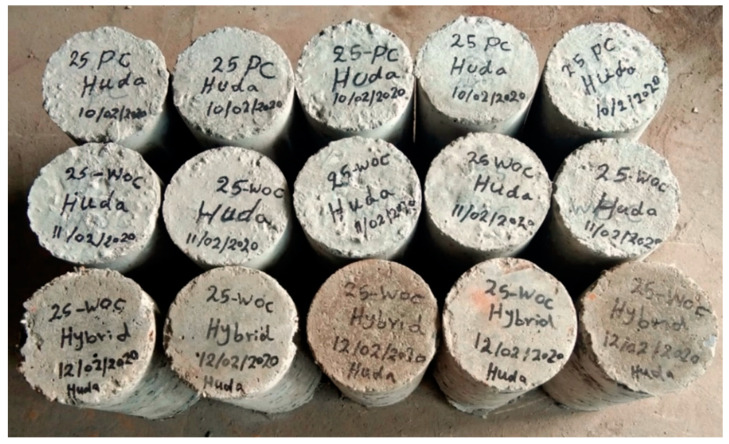
The used concrete specimens.

Overall, 21 cylindrical specimens with a diameter of 150 mm and a height of 300 mm were cast in three groups for testing under high temperatures, as shown in Figure 5; groups are shown in Table 3.

For the present study’s purposes, 21 cylindrical specimens, each 150 mm in diameter and 300 mm in height, were cast in three groups.

First group: based on test methods per IS 10262 guidelines [25], plain concrete (PC) was prepared with natural aggregate (coarse and fine), ordinary Portland cement (OPC 43 grade) with a 0.5 water-cement ratio to be heated in a furnace at 100 °C, 200 °C, and 300 °C.

Second group: based on experimental results of Najm and Ahmad [7], waste ceramic optimal concrete (WOC) was obtained by replacing natural coarse aggregate with 20% ceramic aggregate, fine natural aggregate with 10% ceramic sand, and 43 grade OPC with 10% ceramic powder to be tested at 100 °C, 200 °C, and 300 °C.

Third group: based on experimental results of Najm and Ahmad [26], Hybrid-WOC was obtained by adding 1% PVA fibre and 1% CR-steel fibre with waste ceramic optimal concrete (WOC) to be heated to 100 °C, 200 °C, and 300 °C in a furnace. Designations for various types of concrete are shown in Table 4.

### 3.3. Concrete Mix Design

The concrete mixture for reference specimens containing natural aggregate and ordinary Portland cement (43 grade) was designed for compressive strength of 25 MPa. The water quantity (190 kg/m^3^) and W/C ratio (0.5) were the same for all the concrete mixes. However, they differed in their cement and aggregate (coarse and fine) content because either waste ceramic was used as partial replacements for cement, coarse, and fine aggregates. The quantities of ceramics material, natural material, and fibres used are reported in Table 5. A_WC_, S_WC_, and C_WC_ represent coarse ceramic aggregate, fine ceramic aggregate, and ceramic powder weight per cubic meter of concrete. The mix proportions for different types of concrete are shown in Table 5.

### 3.4. Test Procedures

The most common method to elevate concrete fire resistance is by heating the furnace’s specimens for 28 days. The specimens were heated in normal conditions at a high temperature in the current study. The high-temperature electric furnace (Sigma Scientific Products, Tamil Nadu, India) (Figure 5) was fabricated to test concrete specimens at an elevated temperature of 1150 °C. The inner size of the furnace was 1000 × 760 × 510 mm. The refractory coating was applied to all six sides of the furnace, with the heating elements attached to the left and right sides and the furnace’s top.

The colour changes of PC, WOC, and Hybrid-WOC concrete were determined using the 21 cylinders measuring 150 mm × 300 mm (diameter x height). After 24 h, these specimens were demoulded and cured for 28 days. The specimens were prepared for testing at a single heating-cooling cycle from ambient temperature and exposed to an elevated temperature ranging from 100 °C to 300 °C at an interval of 100 °C after the submerged curing process. The researcher used 300 °C as a maximum range of elevated temperature due to the high water–cement ratio used in the mixed design (0.5 W/C), and this would cause damage to the furnace if we increased the temperature by more than 300 °C.

**Figure 6:**
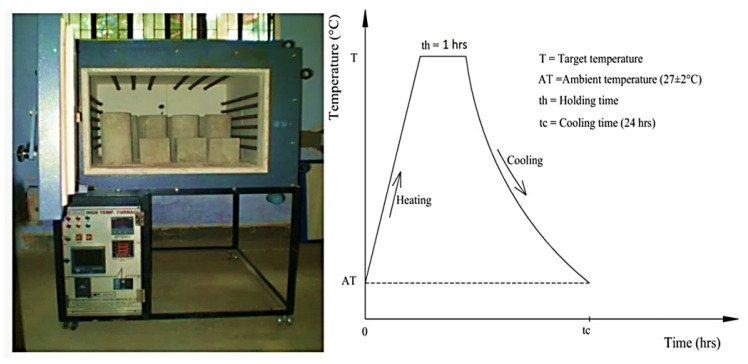
(**a**) High-temperature furnace; (**b**) single heating–cooling cycle curve.

To avoid the cracking of concrete due to high temperature gradient between the core and outside surface, the concrete specimens were heated at a slow rate according to IS:1642 [27]. Otherwise, the cyclic thermal loading would result in a false response. When concrete is suddenly exposed to high or low temperatures, it expands or contracts rapidly, causing disintegration of the material. Thus, the cooling duration was a long time (approximate 20 h) as against the short heating duration (approximate 3 h). This is based on the thermal conductivity of the specimens, their section dimensions, and the peak temperature. Therefore, automatically after heating up to the target temperature (100 °C, 200 °C, and 300 °C) at an average rate of 5 °C/min, (ramp rate 1–2 h), the temperatures were on hold for 1 h and then it was automatically closed down which is shown in Figure 6b, after the cooling process stander ramp downtime 20 h. The alterations in the concrete specimens’ physical characteristics were observed in colour.

### 3.5. Colour Measurement Method

Concrete samples can now be photographed under the same lighting conditions using flatbed scanners (canon lide220, Delhi, India), as proposed by previous studies [28]. These observations can be made without using expensive measurement equipment or computer software for colour analysis. This technique utilises an all-purpose flatbed scanner to capture images, then analyses with image analysis software (Scion Image, version 4.0.3, Scion Corporation, Frederick, MD, USA).

In addition, Hager, I. [29] proposes two different methods for determining concrete’s post-fire exposure temperature using colour analysis. First, the element’s external surface can be examined, which entails keeping an eye on the element’s outer walls (particularly the cement paste). The identification of the temperature field can be assessed by examining the extent of change in the element’s surface colour. A smoke- or soot-covered surface will prevent this method from working.

As an alternative, the visible aggregate on the concrete surface could be observed. The cut surface that results from coring or sawing was examined. Colours can change dramatically due to these processes, revealing aggregate grains that often turn red or pink [21]. This method can also be used on concrete element surfaces that have been damaged by spalling or flaking. In the same regard, Colombo, M. and Felicetti, R. [23] used non-destructive techniques such as digital camera colourimetry for colour change in concrete for scanning the inside concrete surface at elevated temperature; they were found to be sensitive and powerful techniques for evaluating the damages and the colour changes due to fire.

## 4. Results and Discussions

### 4.1. Colour Analysis

Understanding colour and its measurement can be considered complex (see reference [30]) and a limited description is given here. Several colours can be quantified and described in terms of their component by the usage of what is known as colour spaces. There are different forms of these, but they can, more or less, be divided into two sets which denote definite colours either as combinations of other colours, for example, the primary colours red, blue, and green, or in terms of hue, intensity, and saturation.

The mentioned primary colours space is commonly used in monitors and cameras since it is good and simple for displaying and generating images. Separate signals for these colours are gained and then the resultant colour is defined by the percentage of blue, green, and red that are present as its elements. People do not think of the colours red, blue, and green, which is considered a disadvantage of this system.

**Figure 7:**
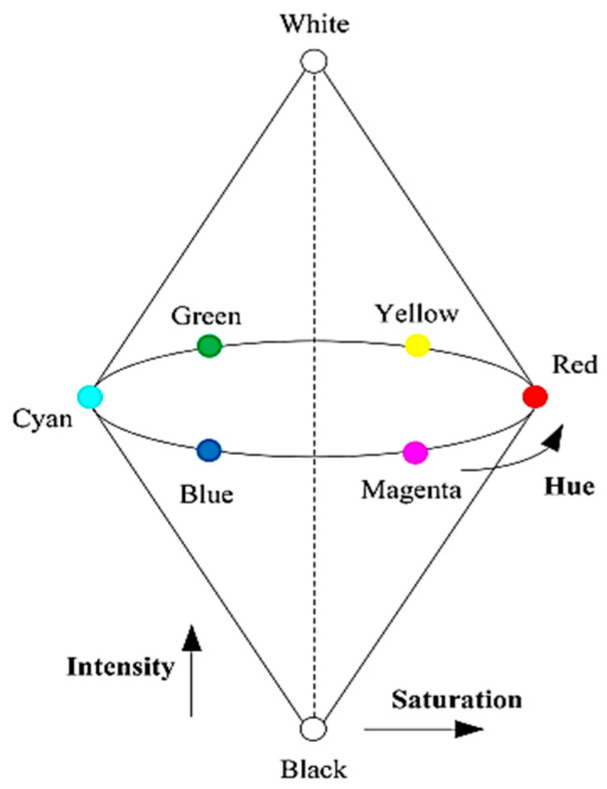
HSI coordinates.

In the hue, saturation, and intensity colour space: hue refers to the kind of colour whether it looks green, yellow, red, etc.; it is the characteristic by which the eye discriminates several parts of the spectrum. It is measured in terms of wavelength. It can be represented by its position on a horizontal circle (see Figure 7). The zero in the circle relies on the measuring of the used system. In the present study, the instrument analyser may quantify the hue as an angle and explain pure red as the zero. Nevertheless, for computer purposes, the pure red is 0 and 255 because the 0–360 range is re-scaled to fit a 0–255 instrument display. Saturation denotes the degree to which a pure colour has diluted with white; for example, highly saturated indicates a bright deep red not diluted with any white. As white is added, the colour changes to pink, to light red then white, completely de-saturated.

In the hue, saturation, and intensity colour space, the circle circumference denotes 100% saturation with 0% at the centre (Figure 7). Intensity is considered a neutral colour value. It represents the degree to which a material reflects light; i.e., it defines the relative darkness or brightness. Consequently, in bright light, red has a high intensity but as light reduces, the intensity decreases and red gets darker and darker until it disappears to black.

Moreover, in the hue, saturation, and intensity colour space, it is denoted by an axis vertical to the circle plane (Figure 7). The full model is accordingly denoted by a specific colour and a double cone is defined in terms of hue, saturation, and intensity by its spot on the cone surface. Samples were saturated with resin, polished, ground, and cut for examination in reflected light.

### 4.2. Colour Change in the New Generation Concrete (WOC and WOC-Hybrid Fibres)

The damage assessment of concrete after heating usually begins with observing the mechanical properties of the concrete, colour changes, and concrete surface cracking. At 100° C temperatures, the specimens’ appearance and colour were the same as those at ambient temperature, with a light grey colour. At 200 °C temperatures, the specimens were grey. Finally, at 300 °C temperatures, the specimen turned to a yellowish-grey colour. The same results were observed by Guo, Y. C. et al. [4].

The physical and chemical transformations in concrete cause the color change in concrete after heating. The cement and fine aggregate components are primarily responsible for the colour change. The whitish colour in concrete after heating is due to the process of dehydration and formation of lime in the cement component. On the other hand, the red colour in concrete after heating is due to the high amount of iron-bearing minerals in the river sand (fine aggregate). Due to the combination colour of cement (whitish colour) and fine aggregate (red colour), the light peach colour of concrete is formed during the heating.

**Figure 8:**
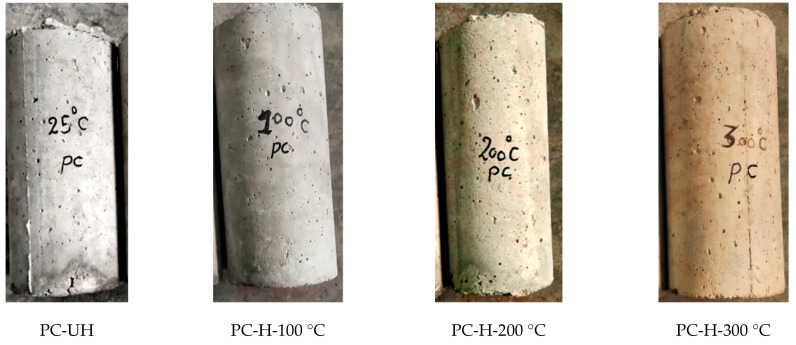

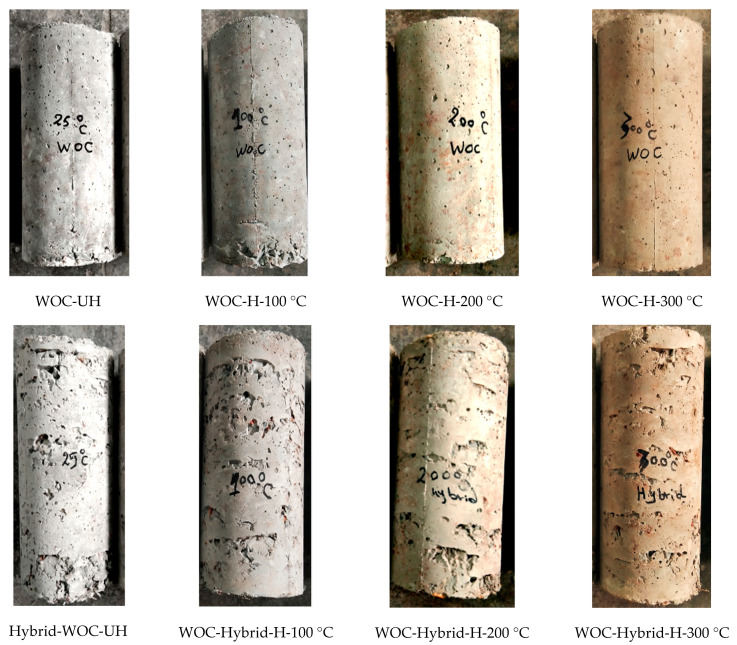
PC, WOC, and WOC-Hybrid specimen colour after being exposed to 100 °C, 200 °C, and 300 °C.

Further, Short, N. R. et al. [21] observed that the concrete specimen’s surface area had a maximum depth for the yellowish-grey colour of 30 mm and disappeared at more than 45 mm. This transition of colour changes was measured when the temperature was less than 300 °C. It was also observed that there was little change observed in the colour of coarse aggregates because aggregates are themselves a product of high temperature and pressure conditions. Figure 8 shows the images of four concrete surfaces, 25 °C, 100 °C, 200 °C, and 300 °C.

In this regard, Colombo, M. et al. [23] observed the colour change in the concrete specimen under the effect of elevated temperatures where it was concluded that concrete’s colour is expected to change from normal to pink or red (300–600 °C), then whitish-grey (600–900 °C), and finally buff at higher temperatures (900–1000 °C). This colour change is more pronounced in siliceous aggregates and less so in calcareous and igneous aggregates, depending on the aggregate type. Detecting the colour change is important because it usually occurs at the same time as the concrete begins to lose strength as a result of heating.

### 4.3. The Mechanism of PC, WOC, and Hybrid-WOC Concrete at Normal Temperature

**Figure 9:**
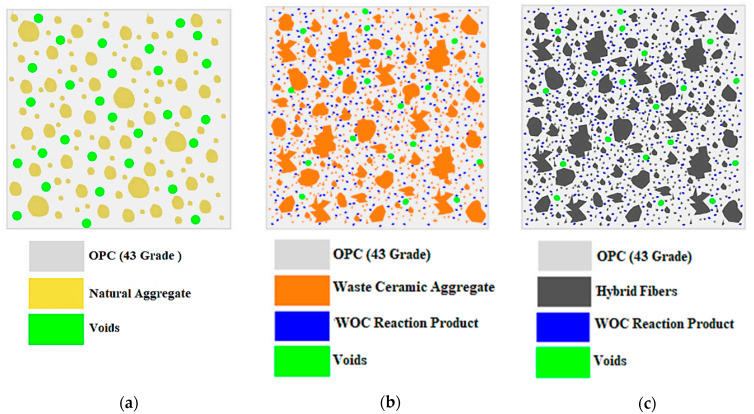
Conceptual diagram of the cross-section of (**a**) PC, (**b**) WOC, and (**c**) WOC-Hybrid concrete (dimensions are not to scale).

Figure 9 depicts conceptual diagrams of the cross-sections of PC, WOC, and WOC-Hybrid, which were drawn to help clarify the mechanism of strength. Hydration, micro-filter, and pozzolanic could all play a role in the process of building strength. When using natural materials, the hydration effect was the primary factor in increasing strength, and this effect persisted for up to 28 days.

As previously stated, the WOC contained a significantly higher percentage of excellent particles, which filled the microvoids in the concrete made with ceramic materials (i.e., lower porosity). Consequently, the hydration and micro-filtering effects contributed to the growth in strength. Furthermore, due to the tiny size of WOC particles, they may have enough specific surface area to allow the pozzolanic reaction.

Finally, in the case of concrete made with hybrid fibre and waste ceramic (WOC-Hybrid), the strength development is due to the PVA fibres effectively controlling the crack propagation at the bending crack tip. The steel fibres resist the widening of the crack width, thereby greatly enhancing the concrete’s strength.

In the presence of water in the mortar matrix, WOC and WOC-Hybrid may react with water-hydrated products of the clinker to form secondary calcium silicate hydrate (CSH) gels. CSH gels would fill the micropores, thereby strengthening the interfacial transition zone (ITZ) and increasing the mortar mix’s strength.

### 4.4. The Mechanism of PC, WOC, and Hybrid-WOC Concrete at Elevated Temperature

According to Netinger, I. et al. [3], concrete with ceramic tiles has lower thermal conductivity than concrete with natural aggregates, which might explain WOC mixtures’ better fire resistance in this research. In addition to using ceramic material to improve heat resistance, hybrid fibres (metallic and non-metallic) and ceramic concrete show very high resistance under elevated temperatures (below 400 °C). Thus, the concurrent use of these two materials in concrete appears to reduce environmental pollution and cost and enhance concrete’s mechanical properties exposed to high temperatures.

Keshavarz, Z. and Mostofinejad, D. [31] concluded that “the positive features of ceramic are high physical strength as well as resistance to freezing and heating. As a result of these features, ceramic tiles do not deform under fire, do not change in colour over time”.

Generally, the addition of non-metallic fibres (PP or PVA) has no significant effect on improving concrete’s CS after exposure to elevated temperature. However, such improvement is visible to a certain extent when FS and TS are considered, particularly at a temperature below 400 °C. Non-metallic fibres (PP or PVA) can increase concrete resistance to cracking, improving its behaviour under the TS test. However, Peng, G. F. et al. [32] prove that “the melting and ignition points of non-metallic fiber are around 150–400 °C. So, the strength of fiber-reinforced concrete decreases when the temperature is above 400 °C due to melting up non-metallic fibers leaving the pores acting at a disadvantage for concrete under any test”.

Han, C. G. et al. [16] noted that “the non-metallic fibers reinforced concrete has much better resistance to thermal spalling than the concrete without fibers because of the melting and ignition of non-metallic fibers. Consequently, the pores formed to expand to form micro-cracks, connecting the existing capillary pores to provide channels for water vapor to escape”. Therefore, an optimum dosage of non-metallic fibres (PVA or PP) around 0.5–1% by the mix’s volume is recommended for concrete to obtain high-temperature resistance.

**Figure 10:**
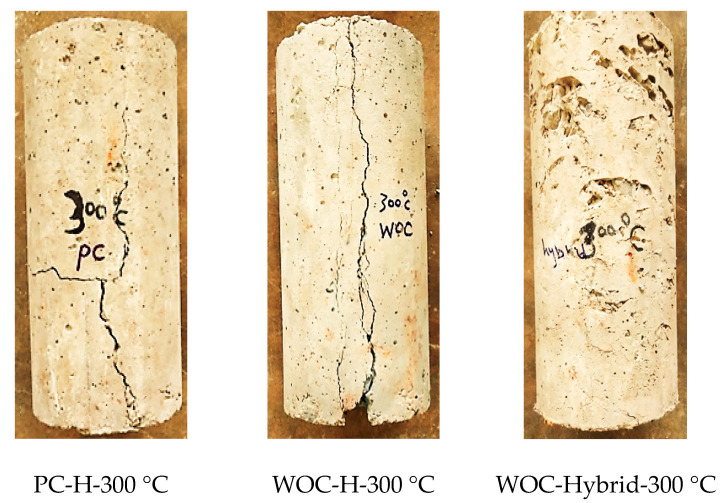
PC, WOC, and WOC-Hybrid cylinder at failure after exposure to elevated temperatures 300 °C.

**Figure 11:**
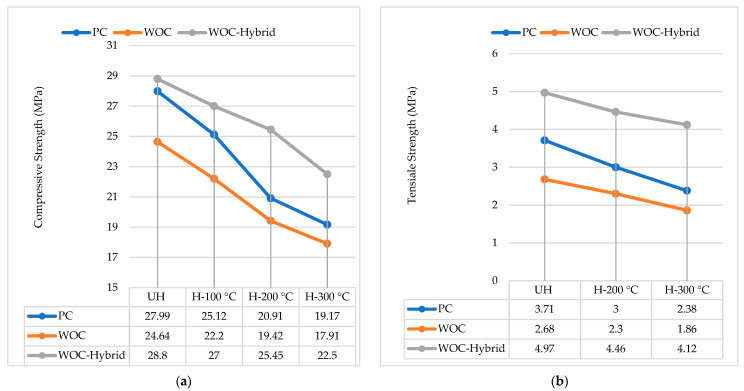
Average (**a**) compressive (**b**) tensile strength for various mixes.

On the other hand, adding metallic fibres (HK or CR) can improve the concrete mechanical properties at elevated temperatures when CS, FS, and TS are considered. These improvements may result from the testing temperatures not being high enough to melt steel fibre. Furthermore, Gao, D. et al. [14] concluded that “metallic fiber has higher thermal conductivity than ordinary concrete materials such as aggregate and cement. Consequently, heat can transmit more uniformly in the fiber-reinforced concrete to reduce the cracks caused by a thermal gradient in concrete, improving concrete performance under compressive & tensile strength test”. Lastly, the resistance to elevated temperatures provided by metallic fibre is weaker than that provided by non-metallic fibre because of the reduced thermal gradient. It is evident by Ahmad et al. [33] that three factors contribute to the deterioration of concrete exposed to high temperatures: physicochemical changes in the cement paste, aggregates, and their thermal incompatibility. So, construction and demolition materials have been discovered to be a good alternative to solving concrete deterioration on a global scale [34]. Figure 10 shows PC, WOC, and WOC-Hybrid cylinder failure after exposure to elevated temperatures, 300 °C. The mechanical properties of PC, WOC, and WOC-Hybrid concrete were measured at ambient temperature and after heating at elevated temperatures for all the concrete specimens as per the guidelines of IS: 516 [35] and as shown in Figure 11.

## 5. Conclusions

This study provided insight into the use of ceramic wastes as partial replacement of cement and natural aggregates in concrete. The effects of elevated temperatures on the physical and mechanical properties of concrete made with ceramics (WOC) as well as those of concrete made with hybrid fibres (WOC-Hybrid) were evaluated. According to the findings, WOC and WOC-Hybrid concrete outperform standard concrete at high temperatures. Therefore, it is recommended to use these types of local and waste materials in buildings in fire-prone areas. Moreover, ceramic waste can be used to make concrete rather than dumping it in landfills, which pose a risk to the environment’s health and sustainability.

Lastly, the relationship between the temperature changes and the accompanying colour changes in the concrete exposed to different high heating temperatures was studied. According to the test results, analysis, and discussions presented in this paper, the following is concluded:

(a) The colour change is mainly because of chemical and physical changes in fine aggregates and cement. At 100 °C, the colour and appearance were similar to those at ambient temperature (light grey), and at 200 °C, the examined specimens’ colours were changed to grey, while at 300 °C, specimens became a yellowish-grey colour.
(b) Among all the tested specimens, hybrid fibre concrete performed better than waste ceramic optimal (WOC) and plain concrete (PC) due to the increase in the transport properties of heated material. In addition, heat transmits more uniformly in the concrete reinforced with hybrid fibres than the other examined types.

In this research, efforts were made to create a new generation of concrete with waste ceramic tiles where it was found that the mechanical and physical properties of concrete would not be harmed if these conventional materials were substituted for waste ceramic ones. Since there are numerous environmental issues that arise from the accumulation of solid waste, the only positive solution is reusing these materials in order to maintain the rapidly depleting environment. Overall, the use of wastes and local materials such as ceramic waste floor or wall tiles remains a positive solution to the sustainability issue of depleting natural aggregate sources.

## Figures and Tables

**Table 1:** Properties of Used Material.

Physical Properties	Cement—OPC	NCA	Sand	C_WC_	A_WC_	S_WC_
Normal consistency (%)	30	–	–	8	–	–
Specific gravity	3.6	2.55	2.6	2	2.35	2.26
Initial setting time	42 min	–	–	54 min	–	–
Final setting time	600 min	–	–	680 min	–	–
7 days compressive strength	21.1 MPa	–	–	37	–	–
Fineness modules	1.4	6.99	2.65	34.1	6.98	2.2
Maximum size	–	0.02 m	–	75 µm	0.02 m	–
Density (kg/m^3^)	3015	618	319	2570	1325	218
Water absorption (%)	–	0.25	1.6	–	4.5	2.52
Crushing value (%)	–	2.86	–	–	4.33	–
Impact value (%)	–	20	**–**	**–**	24.2	–

NCA: natural coarse aggregate, AWC: waste ceramic aggregate, SWC: waste ceramic sand, CWC: waste ceramic cement.

**Table 2:** Chemical analysis of C_WC_ and cement (OPC 43).

Materials	Waste Ceramic Powder (C_WC_)	Cement (OPC 43)
SiO_2_	68.85	22.18
Al_2_O_3_	17	7.35
Fe_2_O_3_	0.8	3.83
CaO	1.7	63.71
Na_2_O	–	0.28
K_2_O	1.63	0.11
MgO	2.5	0.95
TiO_2_	0.737	0.13
MnO	0.078	0.04
LOI	1.78	1.6

**Table 3:** Properties of used fibres.

Fibre Type	CR	PVA
Surface	Plane	-
Cross-section	Circular	-
Anchorage	Continuous	Straight
Length (mm)	50 mm	12 mm
Diameter (mm)	1.14 mm	0.04
Aspect ratio	44	300
Density (g/cm^3^)	7.85	1.3
Tensile strength (MPa)	1242	1560
Elastic modulus (GPa)	200	41

**Table 4:** The total used symbols in the present study.

Symbols	Nomenclature
25PC	M25 plain concrete
25PC-UH	M25 unheated plain concrete
25PC-H-100 °C	M25 heated plain concrete under temperature 100 °C
25PC-H-200 °C	M25 heated plain concrete under temperature 200 °C
25PC-H-300 °C	M25 heated plain concrete under temperature 300 °C
25WOC	M25 waste ceramic optimal concrete
25WOC-UH	M25 unheated waste ceramic optimal concrete
25WOC-H-100 °C	M25 heated waste ceramic optimal concrete under temperature 100 °C
25WOC-H-200 °C	M25 heated waste ceramic optimal concrete under temperature 200 °C
25WOC-H-300 °C	M25 heated waste ceramic optimal concrete under temperature 300 °C
25WOC-Hybrid-UH	M25 waste ceramic optimal hybrid fibre concrete
25WOC-Hybrid-H-100 °C	M25 heated waste ceramic optimal hybrid fibre concrete under temperature 100 °C
25WOC-Hybrid-H-200 °C	M25 heated waste ceramic optimal hybrid fibre concrete under temperature 200 °C
25WOC-Hybrid-H-300 °C	M25 heated waste ceramic optimal hybrid fibre concrete under temperature 300 °C

**Table 5:** Mix proportion.

Mix Ingredients (kg/m^3^)			
Material	PC	WOC	Hybrid-WOC
Water	190	190	190
OPC (43 grade)	380	342	342
C_WC_	-	38	38
NCA	1118	894	894
A_WC_	-	224	224
Sand	609	548	548
S_WC_	-	61	61
Weight Proportion Fibre (by Volume of Concrete)			
PVA-1%	-	-	13
CR-1%	-	-	78

## Data Availability

The data used to support the findings of this study are included within the article.
